# Anti-Oxidative Abilities of Essential Oils from *Atractylodes ovata* Rhizome

**DOI:** 10.1093/ecam/neq006

**Published:** 2011-06-08

**Authors:** Kun-Teng Wang, Lih-Geeng Chen, Duen-Suey Chou, Wen-Li Liang, Ching-Chiung Wang

**Affiliations:** ^1^School of Pharmacy, College of Pharmacy, Taipei Medical University, Taipei 110, Taiwan; ^2^Graduate Institute of Biomedical and Biopharmaceutical Sciences, College of Life Sciences, National Chiayi University, Chiayi, Taiwan; ^3^Graduate Institutes of Pharmacology and Medical Sciences, Taipei Medical University, Taipei, Taiwan

## Abstract

The rhizome of *Atractylodes ovata* De Candolle is rich in essential oils, which are usually removed by processing. In this study, anti-oxidative abilities of essential oils and aqueous extracts of *A. ovata* rhizome were explored, and the influence of processing on the anti-oxidative abilities was examined. Essential oils and aqueous extracts of *A. ovata* were extracted by boiling water and steam distillation, respectively. Quality of these two *A. ovata* samples was controlled by HPLC and GC-MS system, and anti-oxidative abilities were then evaluated. Results showed that surface color of *A. ovata* turned to brown and chemical components were changed by processing. Contents of both atractylon and atractylenolide II decreased in the essential oils, but only the contents of atractylon decreased by processing. Atractylenolide III increased in both *A. ovata* samples. However, *A. ovata* essential oils displayed stronger anti-oxidative abilities than aqueous extracts in DPPH-scavenging, TBH-induced lipid peroxidation and catalase activity assays. Moreover, the bioactivity of essential oils from raw *A. ovata* was stronger than oils from processed *A. ovata*. On the other hand, cytotoxicity of *A. ovata* essential oils was stronger than that of aqueous extracts, and was more sensitive on H9C2 cell than NIH-3T3 and WI-38 cells. In contrast, stir-frying processing method increased cytotoxicity of essential oils, but the cytotoxicity was ameliorated when processed with assistant substances. The results suggested that phytochemical components and bioactivity of *A. ovata* were changed after processing and the essential oils from raw *A. ovata* showed better anti-oxidative and fewer cytotoxicity effects.

## 1. Introduction

Traditional Chinese medicines (TCMs) are widely used to prevent and treat human diseases. However, the pharmacological functions of certain TCMs depend on free radical-scavenging activities [1]. Oxidative stress is associated with the pathogenesis of various diseases. Uncontrolled free radicals can damage myocardial cells, oxidize low-density lipoproteins and eventually result in cardiovascular diseases [2, 3]. In addition, excess reactive oxygen species (ROS) also induce hepatotoxicity and nephrotoxicity in mice [4]. ROS are generated through various pathways, for example, pollutants, UV light and other mechanisms [5].

*Atractylodes ovata* De Candolle is classified in TCMs as a tonic herb. Traditional applications of *A. ovata* were used to invigorate the stomach and spleen by eliminating dampness and to treat gastrointestinal diseases. Many pharmacological effects of the *A. ovata* aqueous extracts (ARE), including anti-inflammation, anti-tumor and immunoregulatory properties have been reported [6–8]. In regard to its chemical constituents, about 0.3–9% essential oils were found in *A. ovata*, including hinesol, *β*-eudesmol, palmitic acid and linoleic acid [9–11]. In addition, atractylon (AT), atractylenolides II (AT-II) and III (AT-III) were the major sesquiterpenoids in the *A. ovata* essential oils (ARO). In our previous study, AT was reported to have strong anti-oxidative abilities [12]. As also previously described, pharmacological effects of ARE were well studied, but the anti-oxidative abilities of the ARO were not very clear.

In TCMs, Chinese herbs are often processed before use. The chemical compositions, efficacy and cytotoxicity of herbs were changed after processing. Different processing methods were employed for TCMs, for example, stir-frying, soaking and carbonizing. *A. ovata* was usually heated and extra essential oils were removed for detoxification before use. Stir-frying with assistant substances (e.g., red soil or burnt clay) was the most popular processing method for *A. ovata* in TCM factories in Taiwan. Red soil and burnt clay are common assistant substances used in modern herbal processing. Red soil contains high concentrations of unhydrated iron oxides, aluminum oxide and heavy clay. Burnt clay is taken from the lining of kitchen stove [13]. Therefore, the anti-oxidative effects of ARE and ARO, as well as whether processing influences the anti-oxidative abilities and cytotoxicity of both samples were explored.

**Figure 1:**
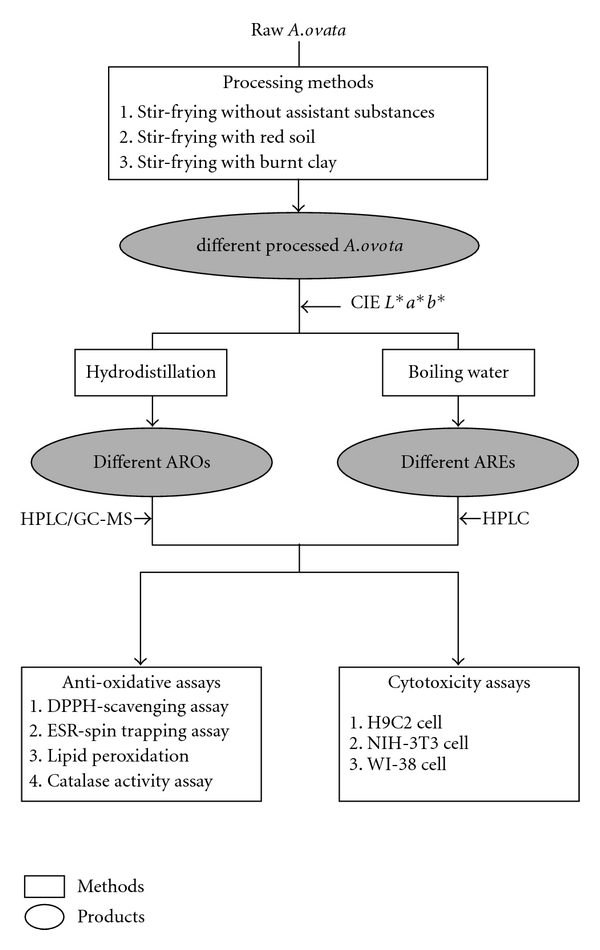
Summary of the experimental procedures of this study. Raw *A. ovata* was processed by different methods, and AREs and AROs were extracted by boiling water and steam distillation, respectively. AREs and AROs were quality controlled by colorimetry, HPLC and GC-MS system and were used to evaluate the anti-oxidative abilities and cytotoxicity effects of *A. ovata*.

In this study, *A. ovata* was processed by different methods and the qualities of processed *A. ovata* were evaluated by colorimetric analysis. ARE and ARO were extracted by boiling water and steam distillation, respectively. The chemical compositions were analyzed by HPLC and GC-MS system. Cytotoxicity and anti-oxidative effects of raw and processed AREs and AROs were also measured by DPPH radical-scavenging, ESR-spin trapping, TBH-induced lipid peroxidation in heart tissue, catalase activity assays and MTT assay, respectively. Our experimental procedure is summarized in Figure 1.

## 2. Methods

### 2.1. Animals

Male Wistar rats weighing about 250 ± 10 g were purchased from the BioLASCO Taiwan Co., Ltd. and kept on a 12:12-h day: night cycle. Animals were maintained in polycarbonate cages at 21 ± 2°C and provided food and water *ad libitum*. All experimental procedures involving animals followed the ethical regulations of Taipei Medical University (Approval No. LAC-97-0122).

### 2.2. Quantitative Colorimetric Analysis of Processed *A. ovata*

The processing procedures of *A. ovata* were described in our previous study [13]. Processed samples were included raw materials, stir-frying without assistant substances for 5 min, stir-frying with red soil for 5 min, stir-frying with burnt clay for 5 min and stir-frying without assistant substances for 30 min. Color measurement of *A. ovata* was performed using the Konica Minolta Color Meter (Model CR-10, Konica Minolta Sensing, Osaka, Japan). The CR-10 was composed of 8 mm diameter measuring area with a diffuse illumination 8° viewing and the color measurements of *A. ovata* were detected on the surface. Each kind of processed *A. ovata* was measured in triplicate. Results were presented as the CIE L*a*b* color system. L* values indicated white or dark samples. A reduction in whiteness, as evidenced by a decrease in L* values, indicates darker samples. The highest a* values and b* values expressed redness and yellowness, respectively.

### 2.3. Preparation and Quality Control of the *A. ovata* Aqueous Extracts

Different *A. ovata* samples were immersed in purified deionized water and boiled for at least 30 min until half of the original amount was left. Aqueous solutions were then filtered, and vacuum freeze-dried. The above preparative procedures were stipulated by the Committee on Chinese Medicine and Pharmacy of Department of Health in Taiwan.

Amounts of AT, AT-II and AT-III were analyzed via HPLC system. HPLC apparatuses were composed of a SCL-10Avp System Controller, an LC-10ATvp Liquid Chromatograph Pump, an SPD-M10A Diode Array Detector, a SIL-10Avp Auto Injector, a CTO-10A Column Oven, FCV-10Avp Flow-Channel Selection Valves (Shimadzu, Tokyo, Japan) and an ERC-3415 Degasser (ERC, Altegolfsheim, Regensburg, Germany). The stationary phase consisted of a Purospher STAR RP-18e reversed-phase column (5 *μ*m, 4 mm i.d × 250 mm, Merck) and an acetonitrile-water system was used as the mobile phase in the gradient mode as follows: acetonitrile: 0–10 min, 40–5%; 10–20 min, 45–55%; 20–30 min, 55–100% and 30–40 min, 100–55%. The flow rate was 1 ml min^−1^, and the oven temperature was maintained at 40°C. We used UV wavelengths of 220 nm to detect the amount of AT and 236 nm to detect the amounts of AT-II and AT-III.

### 2.4. Preparation and Quality Control of the *A. ovata* Essential Oils

*A. ovata* was crumbled and passed through a no. 20 mesh standard sieve. We weighed 70 g of powder samples and mixed them with 5 volumes purified deionized water. Steam distillation of the essential oil was performed in a Clevenger-type apparatus for 7 h according to the Pharmacopoeia of Traditional Medicine in Taiwan. The essential oils were stored in an anhydrous situation. Quality control of the essential oils was analyzed via HPLC and GC-MS system.

The HPLC analysis procedure was modified from our previous study [13]. Analytical instruments were the same as the previous described. The stationary phase consisted of a Purospher STAR RP-18e reversed-phase column (5 *μ*m, 4 mm i.d × 250 mm, Merck) and mobile phase was CH_3_CN-H_2_O (80 : 20, v/v) for AT and CH_3_CN-H_2_O (45 : 55, v/v) for AT-II and AT-III. All mobile phases were degassed by ultrasonication and filtered through a 0.45-*μ*m FP Vericel (PVDF) membrane (Pall Corporation, Ann Arbor, MI, USA). The flow rate was 1 ml min^−1^ and the oven temperature was maintained at 40°C. We used UV wavelengths of 220 nm to detect the amount of AT and 236 nm to detect the amounts of AT-II and AT-III.

The GC-MS system was composed of a gas chromatograph GC-2010 equipped with gas chromatography mass spectrometer GCMS-QP2010 (Shimadzu, Tokyo, Japan). A DB-5MS column (0.25 mm i.d × 30 m × 0.25 *μ*m film, J&W Scientific, Folsom, CA, USA) was performed with Helium as the carrier gas at a constant pressure of 73.0 kPa. Injection temperature and ion source temperature were 150 and 200°C, respectively. The GC oven temperature was programmed to be maintained at 50°C for 5 min and then rise to 250°C at 20°C min^−1^ for 10 min. Results were obtained by collecting the scan mass spectra between the scan range 40 and 800 amu. The mass spectra of the six major peaks in GC-MS chromatogram were compared with the database of NIST/EPA/NIH Mass Spectral Library for identification of possible components.

### 2.5. Anti-Oxidative Assay

#### 2.5.1. DPPH Scavenging Assay

The assay protocol of 1,1-diphenyl-2-picrylhydrazyl (DPPH)-scavenging activities was modified from the previous study [14]. Test samples were dissolved in 100% DMSO and serially diluted into different concentrations. Each sample was mixed with the DPPH ethanolic solution (100 *μ*M) on a 96-well microplate at room temperature for 10 min. Ascorbic acid was used as the positive control. DPPH level of each well was evaluated by detecting the optical density of each well at 530 nm with ELISA spectrophotometer (*μ*Quant, BioTek, USA). The DPPH scavenging rate (%) of each sample was calculated according the following equation: [1 − (*S*_s_/*E_C_*)] × 100, where *S*_s_ and *E*_C_ were the optical density value of the sample and control, respectively.

#### 2.5.2. ESR-Spin Trapping Assay

A Bruker EMX electron spin resonance spectroscopy (ESR) spectrometer was used to detect the ESR trapping and the experimental protocol was modified from the previous report [15]. DPPH radical was used to generate the excess free radical. In sample preparation, DPPH (500 *μ*M) and test samples were thoroughly mixed and reacted for 2 min. ESR spectra were recorded at room temperature using a quartz flat cell designed for aqueous solutions. Dead time of sample preparation and ESR analysis was exactly 30 s after the last addition. Conditions of ESR spectrometry were as follows: 20 mW power at 9.78 GHz, with a scan range of 100 G and a receiver gain of 5 × 10^4^. The modulation amplitude, sweep time, and time constant are given in the figure captions.

#### 2.5.3. Lipid Peroxidation Assay

Heart tissue was obtained from Wistar rats and homogenized in PBS. This homogenized solution was centrifuged for 10 min at 1200 rpm. Protein level in the homogenized tissue was quantified with Bioquant (Merck, Germany). The homogenized tissue was treated with 125 mM *tert*-butylhydroperoxide (TBH) with or without test samples and eventually reacted with thiobarbituric acid (TBA) to form the pink adducts, malondialdehyde (MDA). The optical density was measured at 530 nm with *μ*Quant spectrophotometer (BioTek, USA).

#### 2.5.4. Catalase Activity Assay

The catalase assay protocol was modified from the previous study [16–18]. A discontinuous method for measuring catalase used the ferrous oxidation in xylenol orange solution and detected at 560 nm with *μ*Quant spectrophotometer (BioTek, USA). Catalase activity was represented in units of *μ*mol min^−1^ ml^−1^.

### 2.6. Cytotoxicity Assay

The H9C2 (normal rat cardiomyocytes) and NIH-3T3 (normal mouse embryonic fibroblast) cell lines were maintained in DMEM medium, supporting with 10% fetal bovine serum (FBS) and 10% fetal calf serum, respectively. WI-38 (normal human diploid cell line) cell line was maintained in MEM medium with 10% FBS. When confluent, cells were washed with phosphate-buffered saline (PBS), trypsinized with 0.25% trypsin-EDTA in PBS, washed with fresh culture medium and transferred to 96-well plates (1 × 10^5^ cells ml^−1^) for the cytotoxicity assays. Different test samples were dissolved in DMSO at suitable concentrations and store at –20°C. Test samples were added to each cell line for 24 h without renewal of the medium. The number of surviving cells was then counted using the MTT assay. Finally, the products were evaluated by measuring the optical density of each well at 600 nm with *μ*Quant spectrophotometer (BioTek, USA).

### 2.7. Statistical Analysis

Data are presented as the mean and standard deviation (SD). Significance was calculated using Student's *t*-test and one-way ANOVA test.

## 3. Results

### 3.1. Quality Control of Different Processed *A. ovata*

After being processed by different methods, CIE L*a*b* values of processed *A. ovata* were analyzed (Table 1). Raw *A. ovata* (A) expressed the highest L* values, indicating whitest color. Moreover, stir-frying with red soil for 5 min (C) displayed the highest a* values (redness) and b* values (yellowness), due to the assistant substances, red soil. Finally, we collected 200 pieces of processed *A. ovata* for each group whose CIE L*a*b* values were nearly the same for following assays.

**Figure 2:**
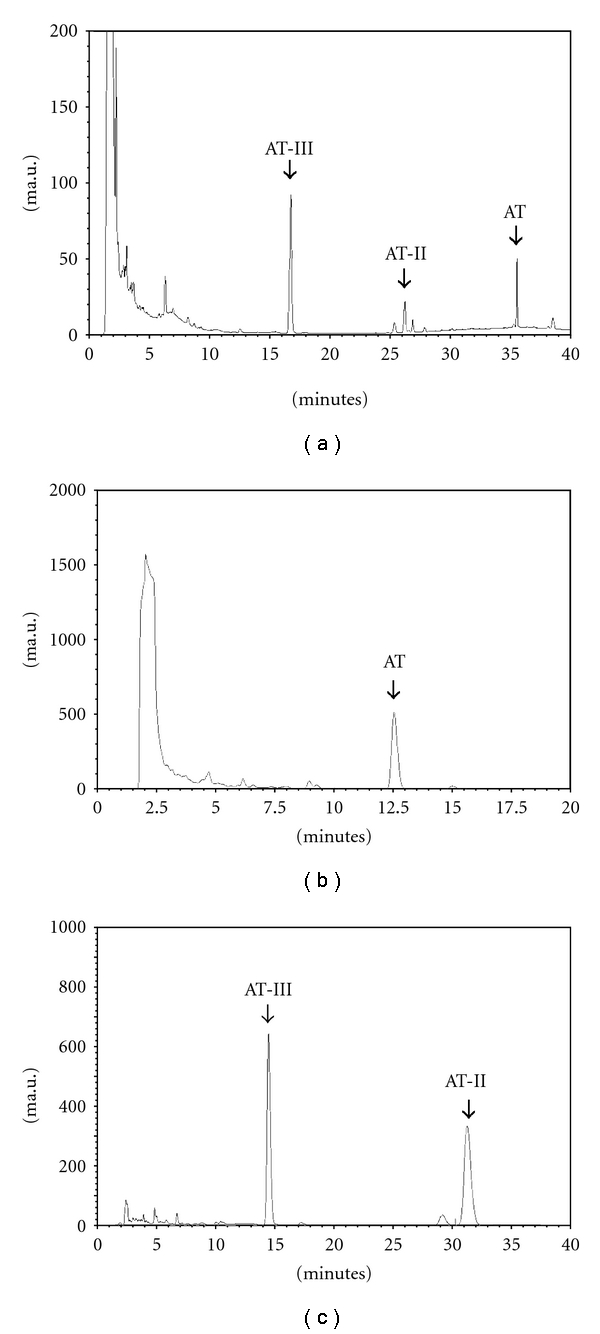
HPLC chromatograms of AT, AT-II and AT-III in AREs (a) and AT (b), AT-II and AT-III (c) in AROs.

**Figure 3:**
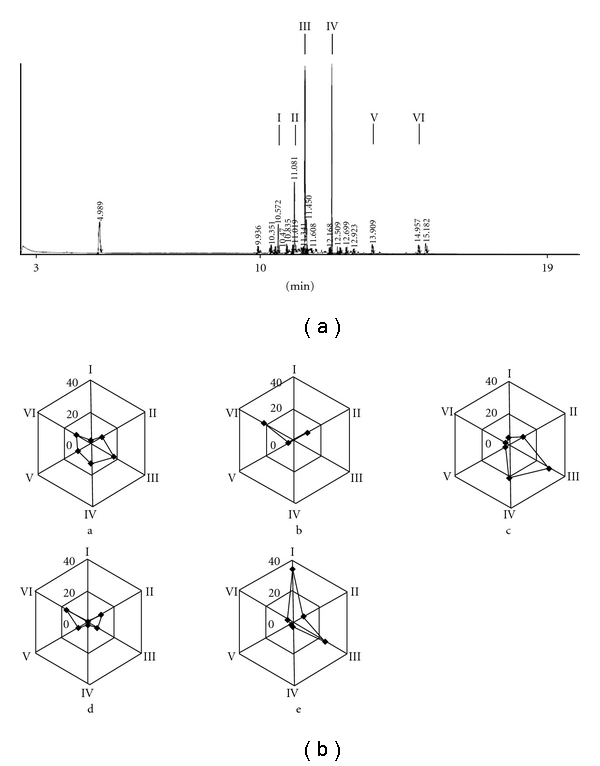
GC-MS fingerprint profile of raw ARO. The arrow indicated the six major components (*β*-caryophyllene, I; *β*-selinene, II; *γ*-selinene, III; AT, IV; AT-II, V; stereoisomer of AT-II, VI) of raw ARO (a). Radar chart analysis of the essential oils from differently processed *A. ovata* (b). a–e indicated the essential oils from raw materials, stir-frying without assistant substances for 5 min, stir-frying with red soil for 5 min, stir-frying with burnt clay for 5 min and stir-frying without assistant substances for 30 min, respectively.

First, the HPLC chromatogram of AT, AT-II and AT-III in AREs was shown in Figure 2(a) and their retention time were 35.5, 26.1 and 16.8 min, respectively. Low concentrations of AT, AT-II and AT-III were found in AREs. Raw *A. ovata* had the highest AT concentration (0.071 ± 0.003 *μ*g mg^−1^) among the different AREs, but it was diminished by stir-frying. After processing, concentrations of AT-II and AT-III in the aqueous extracts from processed *A. ovata* were about 1.5–2.0-fold higher than those from the raw *A. ovata* (Table 2). Secondly, Figure 2(b) displayed the HPLC chromatograms of AT in AROs and its retention time was 12.5 min. Besides, the HPLC chromatogram of AT-II and AT-III in AROs was shown in Figure 2(c) and their retention time were 14.5 min and 32.1 min, respectively. Concentrations of the three sesquiterpenoids in the AROs were higher than those in AREs. The highest AT concentrations (266.70 ± 3.63 *μ*g mg^−1^) were also found in raw ARO. AT and AT-II concentrations were also decreased by processing, but AT-III increased (Table 2) in the ARO. Moreover, the GC-MS fingerprint profile of raw ARO was shown in Figure 3(a). We chose six major components as standard peaks of the AROs. After comparing with the database, their retention time (min) and molecular weight (amu) were *β*-caryophyllene (10.6 and 204), *β*-selinene (11.0 and 204), *γ*-selinene (11.4 and 204), AT (12.3 and 216), AT-II (13.5 and 232) and stereoisomer of AT-II (15.2 and 232), respectively. Compositions of the processed AROs were totally different due to the different processing methods (Table 3). Likewise, concentrations of these six components in AROs were altered by the different processing methods (Figure 3(b)).

### 3.2. DPPH-Scavenging Effects of the *A. ovata* Aqueous Extracts and Essential Oils

DPPH-radical scavenging activities were employed to evaluate the chemically anti-oxidative effects of different AREs and AROs. Firstly, no significant DPPH-scavenging abilities were found in AREs at 5 mg ml^−1^ (data not shown). However, as shown in Table 4, essential oils from raw *A. ovata* (A, 5 mg ml^−1^) displayed the same DPPH-scavenging abilities as ascorbic acid (2.5 mM) and with the IC_50_ value of 2.71 mg ml^−1^. On the other hand, the other samples expressed insignificant DPPH-scavenging effects, and the inhibitory effects were in the following order of essential oils from stir-frying with red soil for 5 min (C, 47.08%), stir-frying with burnt clay for 5 min (D, 38.63%), stir-frying without assistant substances for 5 min (B, 36.87%) and stir-frying without assistant substances for 30 min (E, 33.19%). According to the above results, AROs were evaluated in further anti-oxidative assays.

### 3.3. ESR-Spin Trapping Assay of the *A. ovata* Essential Oils

**Figure 4:**
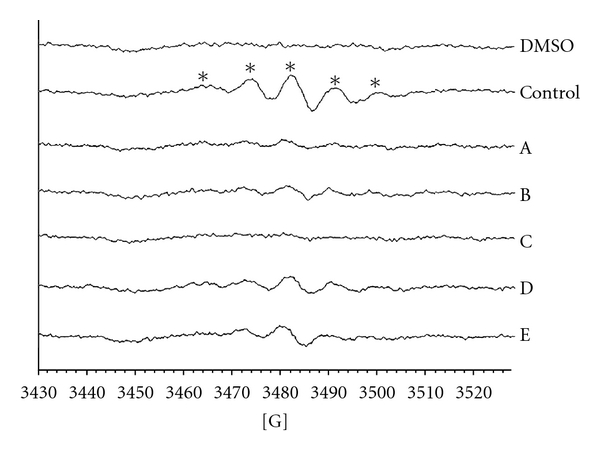
ESR spectra scanning of the anti-oxidative effects of essentials oils from differently processed *A. ovata* samples. The reaction mixture contained 100 mM DMPO and 100% DMSO was added as the solvent control. DPPH (500 *μ*M) was used as the free radical generator, and then the essential oil from raw materials was added, A; stir-frying without assistant substances for 5 min, B; stir-frying with red soil for 5 min, C; stir-frying with burnt clay for 5 min, D; or stir-frying without assistant substances for 30 min, E. Sample concentration was 5 mg ml^−1^. Instrument parameters were as follows: modulation amplitude, 1 G; time constant, 164 ms; scanning for 40 s with three scans accumulated. Asterisk indicate DPPH free radical adduct.

In the ESR-spin trapping assay, DPPH radical was used as the free radical generator. As shown in Figure 4, five major free radical signals were found in the DPPH group. All five different AROs showed inhibitory activity against DPPH free radical generation compared to the DPPH group. Among them, raw ARO (A, 5 mg ml^−1^) displayed the strongest anti-oxidative effect.

### 3.4. Lipid Peroxidative Inhibitory Effects and Catalase Activity of the *A. ovata* Essential Oils

**Figure 5:**
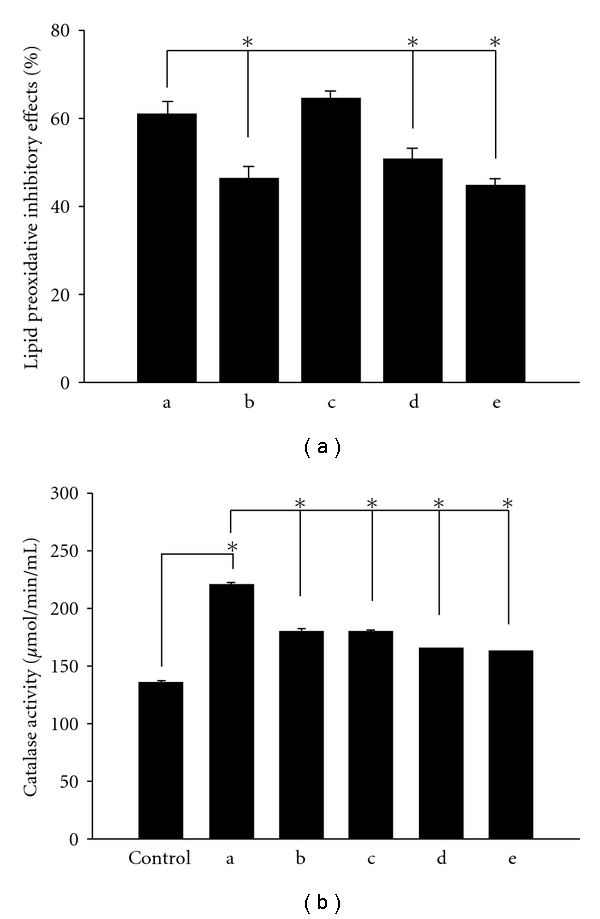
MDA inhibitory effects (a) and catalase activity (b) of essential oils from differently processed *A. ovata* samples. Results are presented as the mean ± SD in triplicate. a–e indicated as the essential oils from raw materials, stir-frying without assistant substances for 5 min, stir-frying with red soil for 5 min, stir-frying with burnt clay for 5 min and stir-frying without assistant substances for 30 min, respectively. Sample concentration was 500 *μ*g ml^−1^. *P < .05.

According to many etiological studies, oxidative stress played an important role in causing many diseases, that is, inflammation and malignant tumor. Thus, the effects of different AROs on TBH-induced lipid peroxidation were evaluated. Essential oils from raw (A, 500 *μ*g ml^−1^) and stir-frying with red soil for 5 min (C, 500 *μ*g ml^−1^) *A. ovata* displayed significant anti-lipid peroxidative activities compared to the other AROs (Figure 5(a)), suggesting that it has stronger MDA inhibitory effects. In addition, all AROs exhibited increased catalase activity. Essential oils from raw *A. ovata* (A, 500 *μ*g ml^−1^) significantly displayed stronger catalase-increasing activity compared to the other AROs (Figure 5(b)).

### 3.5. Cytotoxicity Effects of the Essential Oils and Aqueous Extracts from *A. ovata*

As shown in Table 5, the cytotoxicity effects of AT were stronger than those of AT-II and AT-III, and AT was more sensitive in the H9C2 cell line. In addition, cytotoxicity of AROs on H9C2 cell was also more sensitive than the other cell lines. Firstly, AROs resulting from stir-frying without assistant substances for 5 min (B) and for 30 min (E) displayed stronger cytotoxicity on H9C2 cell line than did raw AROs. Once *A. ovata* was stir-fried with assistant substances, for example, red soil and burnt clay, cytotoxicity effects were ameliorated on H9C2 cell. On NIH-3T3 and WI-38 cell lines, raw ARO showed less cytotoxicity than other AROs. However, none of the aqueous extracts from *A. ovata* showed cytotoxicity effects at 1000 *μ*g ml^−1^ for 24 h on these three normal cell lines.

## 4. Discussion

Essential oils from herbs possessed different bioactivities. For example, essential oils from *Vaccinium corymbosum*, *Garcinia brasiliensis*, *Cyperus rotundus* and *Thymus vulgaris* exhibited effective anti-oxidative activities [19–22]. In addition, the vasorelaxive effect of *Croton nepetaefolius* and anti-fungal activities of *Chenopodium ambrosioides* were due to the essential oils [23, 24]. *A. ovata* was rich in essential oils, but few researches had examined the pharmacological functions of the *A. ovata* essential oils. Only in Zhang's study, essential oils from *Atractylodes lancea* had regulatory effects on delayed gastric emptying in stress-induced rats [25]. Pharmacological characteristics of *A. ovata* were warm, bitter and sweet and commonly used for tonifying and regulating Qi. Doctors usually used *A. ovata* to supple the spleen, boost the stomach, dry dampness and harmonize the center. *A. ovata* could be processed using different methods to meet different clinical needs. Raw *A. ovata* displayed stronger tonifying effects than processed *A. ovata* and was usually used to treat appetite loss, indigestion and tiredness. However, processing enhanced the drying dampness effects of *A. ovata*.

Processing is an important part of preparing Chinese herbs. Characteristics of Chinese herbs are changed through different processing methods. Concentrations of glucosinolates, the active components of Brassica vegetables, decreased after being heated [26]. Variations in chemical composition after processing were correlated to alterations in cytotoxicity and efficacy of Chinese herbs. In Li's study, processing reduced the acute diarrhea effects of Rheum palmatum due to a decrease in the anthraquinone contents [27]. However, maintaining an equal processing level was an important issue. Many reactions occurred during thermal processing of Chinese herbs, like the changes in flavor and color seen after heat treatment. The browning reaction that caused the color changes of Chinese herbs was formed by variations of phytochemical components during heating, resulting in the plant color changes [28]. Prodelphinidin D3 and procyanidin B3, the phenolic compounds of barley grain, were easily auto-oxidized during heating and contributed to browning of the heated barley products [29]. Thus, we controlled the processed level through colorimetric values in this study (Table 1). The different processing techniques changed the various AREs and AROs compositions. In our previous study, we described that AT, AT-II and AT-III were the major *A. ovata* components. Hence, the amounts of AT, AT-II and AT-III in raw and processed *A. ovata* were firstly quantified by HPLC analysis system. In addition, because of the evaporative tendency of the ARO sesquiterpenoids, we used GC-MS system to evaluate the ARO component variations after heat-processing.

In the anti-oxidative assays, AROs displayed stronger DPPH-scavenging effects than AREs (Table 4). Our results showed that raw ARO displayed stronger anti-oxidative abilities than processed AROs in ESR-spin trapping, DPPH-scavenging, TBH-induced lipid peroxidation in heart tissue and catalase activity assays. Changes in the ARO chemical compositions were correlated with their respective anti-oxidative activities. AT was the most potent anti-oxidative components in ROS-induced lipid peroxidation [30]. In stir-frying processing, AT was converted to the non-anti-oxidative components, AT-II and AT-III, and the *A. ovata* essential oils had largely evaporated because of the heating. These phenomenons were possibly the reason that processed *A. ovata* lost its anti-oxidative abilities.

Cytotoxicity effects of AROs and AREs on H9C2, NIH-3T3 and WI-38 cells were also evaluated. The normal rat cardiomyocytes (H9C2) cell line was chosen because of the TBH-induced heart lipid peroxidation assay. In addition, based on the European Centre for the Validation of Alternative Methods (ECVAM, ISO 9001), NIH-3T3 and WI-38 cell lines were used to evaluate the alternative non-animal cytotoxicity tests [31]. As shown in Table 5, AT displayed the strongest cytotoxicity effects among the three sesquiterpenoids. In our previous study, we discussed the variations in chemical components and cytotoxicity between raw and processed *A. ovata* [13]. AT showed the strongest cytotoxicity on different tumor cell line, but AT-II and AT-III did not. However, cytotoxicity effects of the essential oils from raw *A. ovata* which contained the greatest amounts of AT were not stronger than those from processed *A. ovata* on these three normal cell lines. We suggested that although AT was the stronger compound, amounts of AT in the essential oils of raw *A. ovata* (266.70 ± 3.63 *μ*g mg^−1^) was insufficient to cause cell death.

In TCM, the general therapeutical rule of Qi-tonifying herbs was reported to promote blood circulation, activate vital energy circulation and to strengthen the body [32]. Previous study had shown that essential oil played an important role in promoting blood circulation and flow [33, 34]. According to Lou's study, circulatory function correlated to the anti-oxidative activities [35]. In this study, heat processing caused a large loss of *A. ovata* essential oils. We found that loss of essential oils resulted in a reduction of *A. ovata* anti-oxidative effects. Taken together, the cytotoxicity effects of *A. ovata* essential oils were slightly influenced by processing. On the other hand, processing resulted in a large loss of the anti-oxidative abilities of the *A. ovata* essential oils.

**Figure 6:**
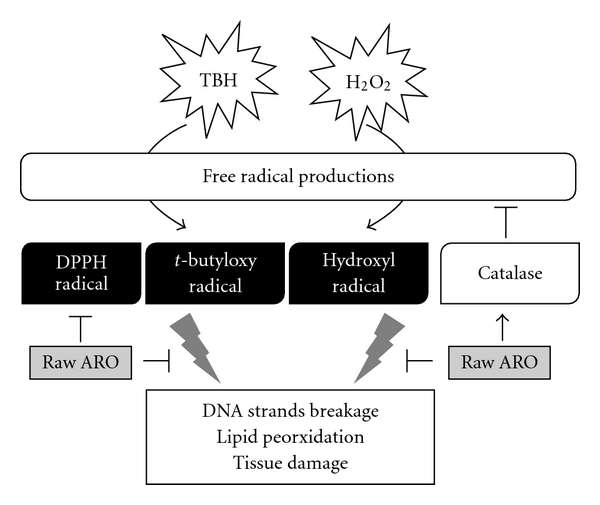
Main effects of raw ARO on oxidative injuries.

In conclusion, *A. ovata* was a potent anti-oxidative Chinese herb whose anti-oxidative effects were decreased by processing. As shown in Figure 6, we suggested that raw *A. ovata* essential oil was a more useful anti-oxidant for its tonifying effects than was processed *A.ovata* essential oil.

## Funding

Committee on Chinese Medicine and Pharmacy, Department of Health, Executive Yuan, Taiwan grants (CCMP96-CP-009).

## Figures and Tables

**Table 1:** Colorimetric values of the differently processed *A. ovata* samples.

	Colorimetric values		
	L*	a*	b*
A	62.3 ± 2.5	9.4 ± 1.1	26.0 ± 2.0
B	53.7 ± 1.7	12.5 ± 0.7	28.7 ± 0.9
C	55.4 ± 1.7	15.7 ± 0.8	31.1 ± 1.1
D	59.4 ± 2.2	7.8 ± 1.1	15.9 ± 1.4
E	24.3 ± 1.6	6.6 ± 1.2	6.7 ± 1.3

Two hundred pieces of *A. ovata* were collected and analyzed in triplicate. Raw materials, A; stir-frying without assistant substances for 5 min, B; with red soil for 5 min, C; with burnt clay for 5 min, D.; without assistant substances for 30 min, E.

**Table 2:** Sesquiterpenoids concentrations of essential oils and aqueous extracts from different processed *A. ovata*.

	Sesquiterpenoids in essential oils (*μ*g mg^−1^)			Sesquiterpenoids in aqueous extracts (*μ*g mg^−1^)		
	AT	AT-II	AT-III	AT	AT-II	AT-III
A	266.70 ± 3.63	113.24 ± 3.01	74.05 ± 1.06	0.071 ± 0.003	0.039 ± 0.001	0.239 ± 0.020
B	203.33 ± 6.64	41.74 ± 1.05	98.70 ± 0.41	0.046 ± 0.004	0.058 ± 0.001	0.297 ± 0.006
C	145.07 ± 3.04	78.43 ± 4.12	92.82 ± 2.72	0.057 ± 0.003	0.061 ± 0.001	0.290 ± 0.006
D	147.51 ± 3.40	49.21 ± 4.64	81.28 ± 1.90	0.008 ± 0.001	0.060 ± 0.001	0.301 ± 0.004
E	95.81 ± 0.77	13.84 ± 0.84	25.98 ± 0.51	0.000 ± 0.000	0.027 ± 0.001	0.053 ± 0.001

Raw materials, A; stir-frying without assistant substances for 5 min, B; with red soil for 5 min, C; with burnt clay for 5 min, D; without assistant substances for 30 min, E.

**Table 3:** Peak area percentage of essential oils from differently processed *A. ovata* samples analyzed by GC-MS.

	Peak area (%)					
	I	II	III	IV	V	VI
A	3.53	13.69	28.16	20.76	15.87	17.99
B	1.72	25.29	4.37	2.06	9.69	56.87
C	6.90	14.94	41.99	29.42	3.10	3.64
D	1.70	23.33	16.89	3.80	16.43	37.84
E	46.36	11.21	32.54	3.00	2.00	4.88

Raw materials, A; stir-frying without assistant substances for 5 min, B; with red soil for 5 min, C; with burnt clay for 5 min, D.; without assistant substances for 30 min, E. The six major components were *β*-caryophyllene (I), *β*-selinene (II), *γ*-selinene (III), AT (IV), AT-II (V), stereoisomer of AT-II (VI).

**Table 4:** DPPH scavenging activities of essential oils from differently processed *A. ovata* samples.

DPPH inhibitory effects		
Sample (mg ml^−1^)	Inhibition^a^ (%)	IC_50_ value
A	63.53 ± 0.25	2.71
B	36.87 ± 0.23	>5
C	47.08 ± 2.88	>5
D	38.63 ± 3.34	>5
E	33.19 ± 0.99	>5
Ascorbic acid (mM)	67.94 ± 0.96	1.22

Concentration of each sample was 5 mg ml^−1^, except ascorbic acid (2.5 mM). Raw materials, A; stir-frying without assistant substances for 5 min, B; with red soil for 5 min, C; with burnt clay for 5 min, D.; without assistant substances for 30 min, E.

**Table 5:** Cytotoxicity effects of sesquiterpenoids and essential oils from differently processed *A. ovata* samples.

	IC_50_ values		
	H9C2	NIH-3T3	WI-38
Sesquiterpenoids			
AT^a^	40.82 ± 4.96	46.71 ± 4.69	62.29 ± 10.11
AT-II	>400	>400	>400
AT-III	>400	>400	>400

Essential oils			
A^b^	200.27 ± 1.11	283.30 ± 9.77	252.73 ± 16.79
B	163.03 ± 2.83^c^	244.35 ± 11.94	218.15 ± 9.29
C	217.92 ± 2.67^d^	241.49 ± 20.69	271.67 ± 14.75
D	266.14 ± 2.65^d^	225.19 ± 3.18	214.21 ± 17.25
E	170.68 ± 1.86^c^	219.96 ± 6.43	247.81 ± 1.05

aUnits of AT to AT-III and A to E were *μ*M and *μ*g ml^−1^, respectively.

bConcentrations of A–E were 1000  *μ*g ml^−1^.

cCompared with A, *P* < .05.

dCompared with A, *P* < .05.

Raw materials, A; stir-frying without assistant substances for 5 min, B; with red soil for 5 min, C; with burnt clay for 5 min, D.; without assistant substances for 30 min, E. Each test was in triplicate.
